# Association between Glucocorticoid Receptor Methylation and Hippocampal Subfields in Major Depressive Disorder

**DOI:** 10.1371/journal.pone.0085425

**Published:** 2014-01-21

**Authors:** Kyoung-Sae Na, Hun Soo Chang, Eunsoo Won, Kyu-Man Han, Sunyoung Choi, Woo Suk Tae, Ho-Kyoung Yoon, Yong-Ku Kim, Sook-Haeng Joe, In-Kwa Jung, Min-Soo Lee, Byung-Joo Ham

**Affiliations:** 1 Department of Psychiatry, Gachon University Gil Medical Center, Incheon, Republic of Korea; 2 Department of Medical Bioscience, Graduate school, Soonchunhyang University, Bucheon, Republic of Korea; 3 Department of Psychiatry, College of Medicine, Korea University, Seoul, Republic of Korea; 4 Brain and Cognitive engineering, Korea University, Seoul, Republic of Korea; 5 Neuroscience Research Institute, College of Medicine, Kangwon National University, Chuncheon, Republic of Korea; University of Utah, United States of America

## Abstract

**Background:**

DNA methylation in the promoter region of the glucocorticoid receptor gene (*NR3C1*) is closely associated with childhood adversity and suicide. However, few studies have examined *NR3C1* methylation in relation to major depressive disorder (MDD) and hippocampal subfield volumes. We investigated the possible association between *NR3C1* methylation and structural brain alterations in MDD in comparison with healthy controls.

**Methods:**

We compared the degree of *NR3C1* promoter methylation in the peripheral blood of non-psychotic outpatients with MDD and that of healthy controls. Correlations among *NR3C1* promoter methylation, structural abnormalities in hippocampal subfield volumes and whole-brain cortical thickness, and clinical variables were also analyzed.

**Results:**

In total, 117 participants (45 with MDD and 72 healthy controls) were recruited. Patients with MDD had significantly lower methylation than healthy controls at 2 CpG sites. In MDD, methylations had positive correlations with the bilateral cornu ammonis (CA) 2–3 and CA4-dentate gyrus (DG) subfields. However, in healthy controls, methylations had positive correlation with the subiculum and presubiculum. There were no differences in total and subfield volumes of the hippocampus between patients with MDD and healthy controls. Compared with healthy controls, patients with MDD had a significantly thinner cortex in the left rostromiddle frontal, right lateral orbitofrontal, and right pars triangularis areas.

**Conclusions:**

Lower methylation in the *NR3C1* promoter, which might have compensatory effects relating to CA2-3 and CA4-DG, is a distinct epigenetic characteristic in non-psychotic outpatients with MDD. Future studies with a longitudinal design and a comprehensive neurobiological approach are warranted in order to elucidate the effects of *NR3C1* methylation.

## Introduction

Major depressive disorder (MDD) is one of the most common psychiatric illnesses [1]. According to the World Health organization (WHO), MDD is expected to be the second leading cause of disease burden by 2030 [2]. Given its increasing socioeconomic impact, numerous studies have investigated possible environmental and genetic factors contributing to MDD. Since MDD has a particularly low heritability rate (0.32) compared to schizophrenia (0.67) and bipolar disorder (0.62) [3], it is likely that gene-environment interactions play an important role in the development of MDD. Although a clear definition has not yet been established, epigenetics generally refers to (possibly heritable) changes in genetic activity without DNA sequence alterations through interactions with environmental factors [4].

The methylation at CpG sites in the promoter region of the glucocorticoid receptor gene *NR3C1* is one of the most widely investigated epigenetic alterations in the field of psychiatry. Glucocorticoid secretion is a primary response to stress, and glucocorticoid receptor dysfunction accompanied by hypothalamus-pituitary-adrenal (HPA) axis hyperactivity has been hypothesized to be one of the major pathophysiologic alterations in MDD [5], [6], although there are some inconsistencies [7], [8]. Weaver et al. (2004) first reported that insufficient maternal licking and grooming in rats resulted in *NR3C1* methylation in the promoter region exon 1_7_, which corresponds to exon F_1_ in humans, and adversely affected HPA axis response to stress [9]. Subsequent human studies have reported that prenatal risk factors or childhood adversity was associated with DNA methylation in the exon F_1_ region of the *NR3C1* in the postmortem hippocampus of suicide victims [10]–[12]. The results suggest that higher methylation in *NR3C1* in relation to early life adversities may also have causative roles for depression, which is closely related to suicide.

However, there have been no studies directly comparing *NR3C1* methylation between patients with MDD and healthy controls. One of the previous studies measured *NR3C1* methylation in patients with borderline personality disorder and MDD, but healthy controls were not included in the study [13]. The results showed that patients with borderline personality disorder, who had a significantly higher rate of childhood abuse, had significantly higher *NR3C1* methylation than patients with MDD. Additionally, most epigenetic studies did not consider possible associations between epigenetic patterns and abnormalities in brain structure, particularly in the hippocampus, where most of the glucocorticoid receptors are located. Furthermore, the functions of the hippocampus vary according to subfields. For example, the dentate gyrus (DG), the cornu ammonis (CA) 3, and the CA4 (also referred to as hilus) are closely associated with treatment response in depression [14], whereas CA1 is the subfield most vulnerable to vascular injury [15]. On the other hand, glucocorticoid receptors are widely present in other brain regions such as the prefrontal cortex, amygdala, and hypothalamus [16], [17]. Glucocorticoid receptors in the forebrain play an important role in depression [6], [18]. Thus, structural analysis of the whole brain, as well as hippocampal subfield volume, should also be conducted to comprehensively identify neuroanatomical associations between *NR3C1* methylation and MDD.

Given the above considerations, we hypothesized that patients with MDD would show higher *NR3C1* methylation than healthy controls, which is consistently shown in studies investigating the association between methylation and childhood adversity. Subsequently, we further investigated the relationships between *NR3C1* methylation and brain structural abnormalities in patients with MDD and healthy controls.

## Methods and Materials

### Ethics statement

All participants were provided with a full explanation of this study and gave written informed consent before enrollment in the study. The study protocol was reviewed and approved by the Institutional Review Board (IRB) of Korea University Anam Hospital. This study was conducted in accordance with the Declaration of Helsinki as revised in 1989.

### Participants

All subjects were aged 18 to 65 and recruited at Korea University Anam Hospital. Edinburgh Handedness Test [19] was applied to all participants before imaging acquisition to determine handedness. Since brain function and structures could be affected by handedness [20], only those who were right-handed were included in this study. Outpatients who were diagnosed with MDD were recruited. An Axis I diagnosis was determined by a board-certified psychiatrist, according to the Diagnostic and Statistical Manual for Mental Disorders (DSM-IV) [21], using the Korean version of the Structured Clinical Interview for DSM-IV (SCID-IV) (Han, 2000). Patients were excluded if they had: (1) a past or present history of comorbid axis I or II disorders according to DSM-IV criteria, (2) psychotic features (3) a history of taking antidepressants (4) an IQ score under 80, (5) a history of primary neurologic diseases, such as Parkinson's disease and epilepsy, (6) organic brain lesions, such as cerebrovascular or space-occupying lesions, or (7) any contraindications for magnetic resonance imaging (MRI) such as pacemakers. Age- and sex-matched healthy controls who were confirmed to have no present, or past history of, psychiatric illnesses by board-certified psychiatrists were enrolled.

Severity of depression and perceived stress for all participants were measured on the day of MRI acquisition. Depression was measured by the 17-item Hamilton Rating Scale for Depression (HRSD) [22]. Perceived stress was evaluated by the perceived stress scale (PSS) [23], which consists of 14 5-point items. The PSS measures stress perceived by patients for the last month. Scores range from 0 to 40, and higher scores represent more stress. The reliability and validity has been established in Korean [24].

### Selection of genomic regions of the *NR3C1* gene for methylation analysis

We measured methylations at 5 CpG sites (CpG1 = −293, CpG2 = −286, CpG3 = −283, CpG4 = −277, and CpG5 = −274 in the 1F regions, distance (nt) from transcription start site (+1)) in accordance with previous studies [13], [25].

### DNA methylation analysis by pyrosequencing analysis

We used the bisulfite pyrosequenicng method for methylation analyses of the *NR3C1* promoter regions. Polymerase chain reaction (PCR) and sequencing primers were designed using Pyrosequencing Assay Design Software v2.0 (Qiagen; Valencia, CA, US). The primer sequence and target region are listed in **Table 1** and **Fig. S1 in File S1**. Bisulfite-modified gDNA was prepared using EZ DNA Methylation-Gold kit (Zymo Research; Orange, CA, US), according to the manufacturer's instructions. The bisulfite reaction was carried out on 500 ng gDNA, and the reaction volume was adjusted to 20 µl with sterile water, and 130 µl of CT conversion Reagent was added. The sample tubes were placed in a thermal cycler (MJ Research, Inc., Waltham, MA, US) and the following steps were performed: 10 min at 98°C, 2 h 30 min at 64°C, and stored at 4°C. The DNA was purified using reagent contained in EZ DNA Methylation-Gold kit (Zymo Research; Orange, CA, US). The converted samples were added into a Zymo-Spin ICTM Column containing 600 µl of the M-Binding Buffer and mixed by inverting the column several times. The column was centrifuged at full speed for 30 s and the flow-through was discarded. The column was washed by adding 200 µl of M-Wash Buffer and spinned at full speed, and then 200 µl of M-Desulphonation Buffer was added to the column and was let stand at room temperature (20–30°C) for 15–20 min. After incubation, the column was centrifuged at full speed for 30 s. The column was washed by adding 200 µl of M-Wash Buffer and spinned at full speed (this step was repeated). The converted gDNA was eluted by adding 20 µl of M-Elution Buffer into the column and spinned. DNA samples were finally stored at −20°C until further use.

**Table 1:** Primers for bisulfite polymerase chain reaction and pyrosequencing.

Genes	Primer (5′–3′)		Assayed CpG sites^a^	Annealing Tm	Amplicon size
*NR3C1*	**Forward**	GGAAGGAGGTAGAGAGAAAAGAAATTG	−293	55°C	175 bp
			−286		
	**Reverse**	Biotin- AACTCCCCAAAAAAAAAAATAAC	−283		
			−277		
	**Sequencing (F)**	TTAAAGTAGTTTTAGAGAGATTAGG	−274		

^a^ Distance (nt) from transcription start site (+1).

PCR was carried out in a volume of 20 µl with 20 ng or more of converted DNA, 2.5 µl of 10× Taq buffer, 5-unit Hot/Start Taq polymerase (Enzynomics; Daejeon, Korea), 2 µl of each 2.5 mM dNTP mixture, 1 µl of 10 pmole/µl Primer-S, and 1 µl of 10 pmole/µl biotinylated-Primer-As. The amplification was carried out according to the general guidelines suggested by Pyrosequencing: denaturing at 95°C for 10 min, followed by 45 cycles at 95°C for 30 s, at 55°C for 30 s, at 72°C for 30 s, and a final extension at 72°C for 5 min. The PCR (2 µl) was confirmed by electrophoresis in a 2% Agarose gel and visualized by ethidium bromide staining. ssDNA template was prepared from 16 to 18 µl biotinylated PCR product using streptavidin Sepharose® HP beads (Amersham Biosciences AB; Upsala, Sweden) following the PSQ 96 sample preparation guide using multichannel pipets. For analysis, 15 pmole of the respective sequencing primer was added. Sequencing was performed in a PyroMark ID system with the Pyro Gold reagents kit (Qiagen; Valencia, CA, US), according to the manufacturer's instructions without further optimization. The degree of methylation at each CpG site was estimated using Pyro Q-CpG™ Software (Qiagen; Valencia, CA, US).

### MRI Acquisition

Three-dimensional structural MRI scans were acquired from a 3.0 T Siemens Trio whole-body imaging system (Siemens Medical Systems, Iselin, NJ, USA), using a T1-weighted magnetization-prepared rapid gradient-echo (MP-RAGE (1900 ms repetition time, 2.6 ms echo time, 220 mm field of view, 256×256 matrix size, 176 coronal slices without gap, 1×1×1 mm, 3 voxels, 16° flip angle, number of excitations = 1).

### MRI processing for cortical thickness and hippocampal segmentation

Cortical thickness, defined as the shortest distance between gray/white matter boundary and the spiral surface at each point across the cortical mantle [26], was automatically estimated by FreeSurfer (software version 5.0, http://surfer.nmr.mgh.harvard.edu), which is the most widely used method [27]. The technical details of these procedures have been described elsewhere [26], [28]. Briefly, the process consists of correction for motion artifact, averaging over multiple T1 images, removal of non-brain tissue, Talairach transformation, white matter and subcortical gray matter segmentation, tessellation of the gray matter-white matter boundary, automatic correction for topological defects, intensity normalization, surface deformation, and parcellation of the cerebral cortex into anatomical regions. Total intracranial volume (TIV) was also automatically calculated by FreeSurfer software [29]. Through careful inspection of all raw images at segmented and inflated stages, we confirmed that no images had substantial defects. For further analysis, cortical maps were smoothed using a Gaussian kernel with a full width at half maximum of 10 mm.

The automated hippocampal subfield segmentation was estimated using a Bayesian model included in the FreeSurfer software package, as suggested in a previous study [30]. The right and left hippocampus were segmented into the following 7 subfields: CA1, CA2-3, CA4-DG, subiculum, presubiculum, fimbria, and hippocampal fissure.

### Statistical analysis

Demographic data were compared between patients with MDD and healthy control groups by chi-square test for dichotomous variables and independent *t*-test for continuous variables. Methylation between patients with MDD and healthy controls were compared using analysis of covariance (ANCOVA) adjusted for age. To prevent inflated positive results (type I error), the significance level was set according to Bonferroni-corrected α values in the between-group analysis (*p*<0.01) when comparing methylation at 5 CpG sites.

Hippocampal subfield volumes and regional cortical thickness were imported into the SPSS 16.0 software (Chicago, IL, US) for statistical analysis. Relationships among hippocampal subfield volumes, methylation, and duration of illness were analyzed.

In the case of cortical thickness, a vertex-wise general linear model, with controlling for age, sex, and total intracranial volume, was used to detect the following: 1) differences in cortical thickness between patients and controls, 2) correlation between CpG methylation and cortical thickness in patients with MDD and healthy controls. Age, sex, and total intracranial volume were included as covariates for all analyses regarding brain structures. Multiple comparisons correction was applied to between-group analyses in hippocampal subfield structures and cortical thickness. Regarding hippocampal subfield analysis, *p*<0.0036 (0.05/14) was applied. Regarding cortical thickness, Monte-Carlo permutation test implemented in FreeSurfer were applied, and the statistical significance level was considered at cluster-wise probability (CWP), which is similar to alpha significance, *p*<0.05. In the case of explorative correlation analyses among methylation, brain structures, and clinical variables, uncorrected p values (*p*<0.05) were applied.

## Results

### Participants

A total of 117 participants (45 with MDD and 72 healthy controls) were recruited. Demographic and clinical data for patients with MDD and healthy controls are presented in **Table 2**. There were no significant differences in age and sex between the 2 groups. Among the MDD group, 39 of 45 MDD patients had a mild to moderate degree of depression, whereas only nine (20%) patients were severely depressed [31].

**Table 2:** Demographic and clinical data for patients with major depressive disorder and healthy controls.

	MDD	HC	*t* or *χ^2^*
**Age**	41.60 (11.80)	40.72 (14.20)	0.346
**Sex, female**	34	51	0.311
**Education (years)**	12.96 (2.80)	13.84 (2.93)	−1.568
**Family history of MDD, yes** *	11	1	15.992
**HRSD scores** *	19.84 (5.68)	2.03 (2.23)	20.10
**PSS scores** *	18.13 (6.77)	12.69 (8.47)	3.83
**Duration of illness (months)**	29.78 (36.51)		
**Number of past depressive episodes**			
**0**	24 (53.3)		
**1**	12 (26.7)		
**2**	4 (8.9)		
**3**	5 (11.1)		

All data are represented as mean (SD) or number (%).

MDD: major depressive disorder, HC: healthy controls, HRSD: Hamilton Rating Scale for Depression, PSS: Perceived Stress Scale.

*p*<0.001.

### Comparison of *NR3C1* methylation and hippocampal subfield volumes between patients with MDD and healthy controls

**Figure 1:**
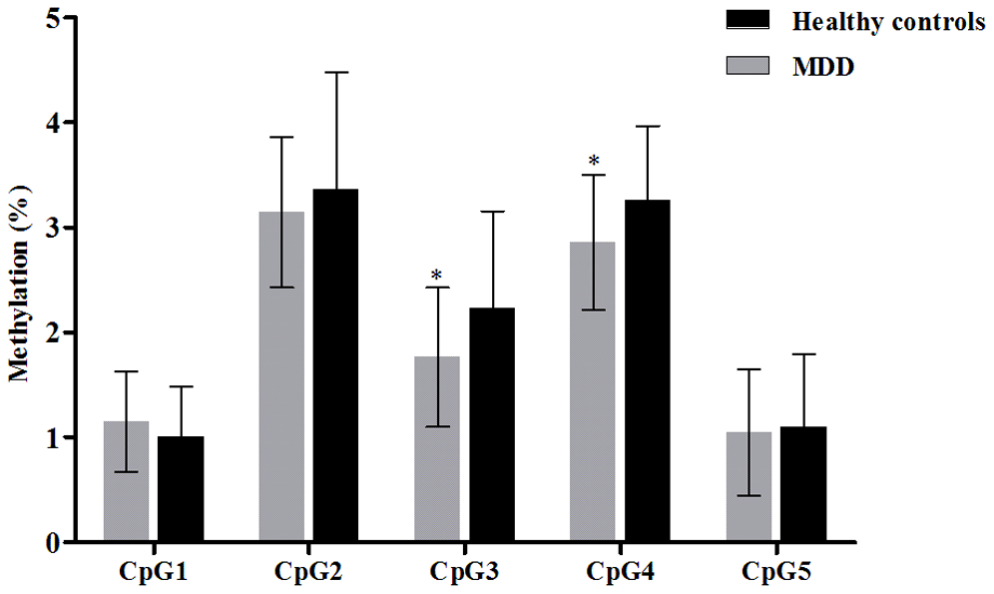
Comparison of *NR3C1* methylation between patients with major depressive disorder and healthy controls, mean (SD). ^*^ *p*<0.01.

**Figure 2:**
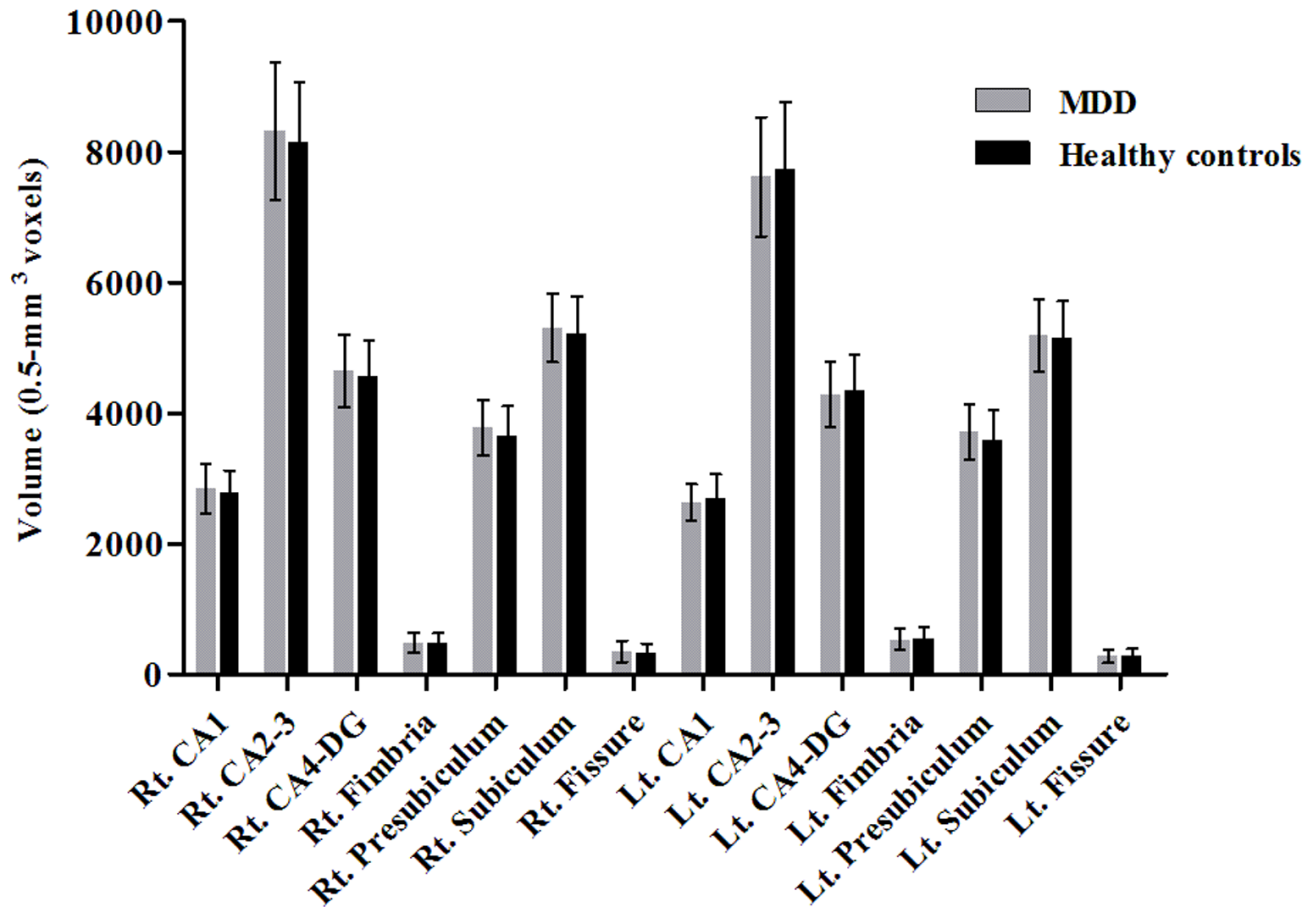
Comparison of hippocampal subfield volumes between patients with major depressive disorder and healthy controls, mean (SD).

Patients with MDD had significantly lower methylation at CpG 3 and CpG4 compared to healthy controls (**Fig. 1** **, Table S1 in File S1**). There were no significant differences in CpG sites 1, 2, and 5 between the 2 groups. There were no differences in hippocampal subfield volumes between patients with MDD and healthy controls (**Fig. 2** **, Table S2 in File S1**).

### Comparisons of cortical thickness between patients with MDD and healthy controls

**Figure 3:**
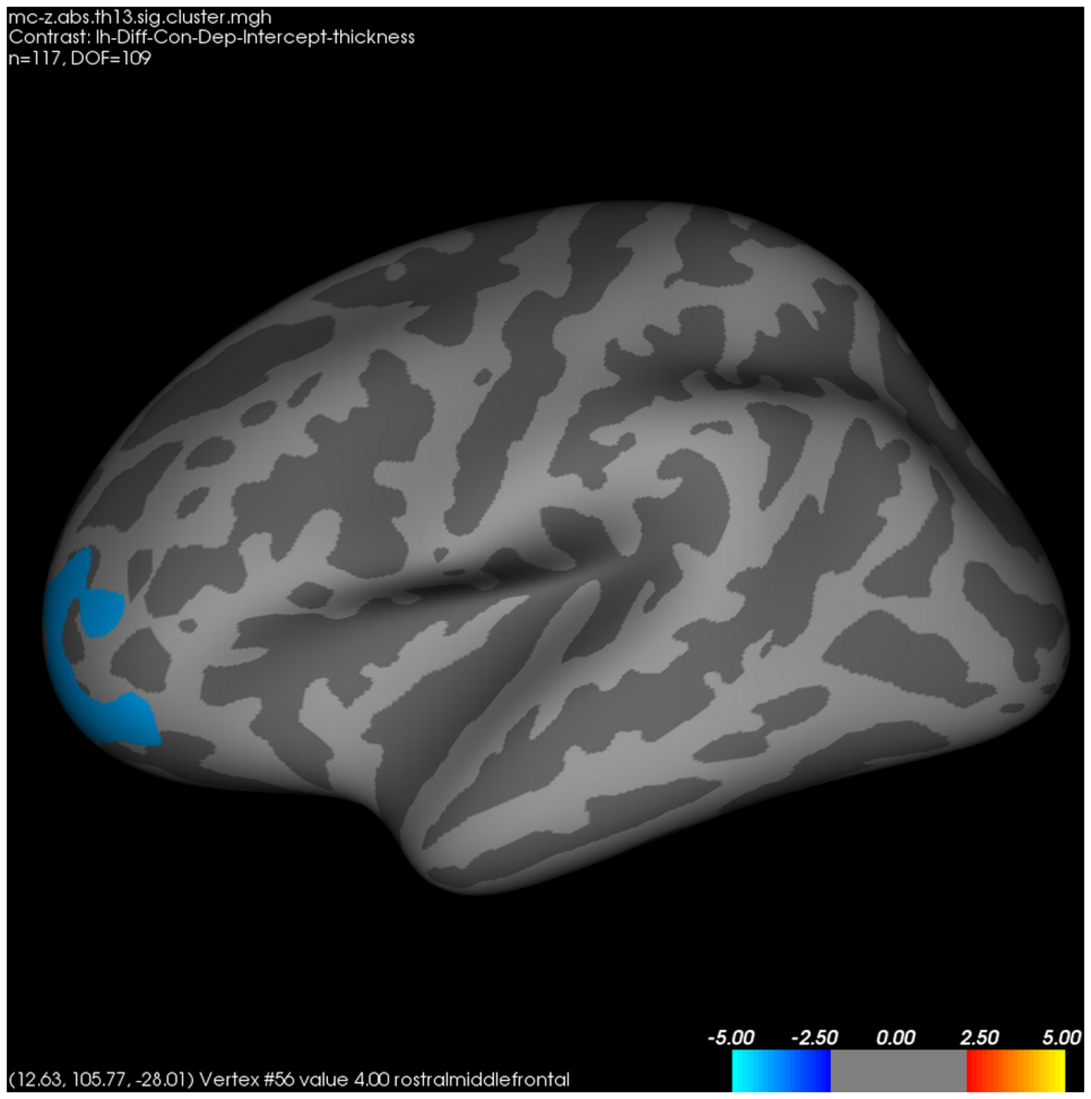
Differences in left cortical thickness between patients with major depressive disorder and healthy controls. *FWE*-corrected *p*<0.05 through Monte-Carlo permutation test.

**Figure 4:**
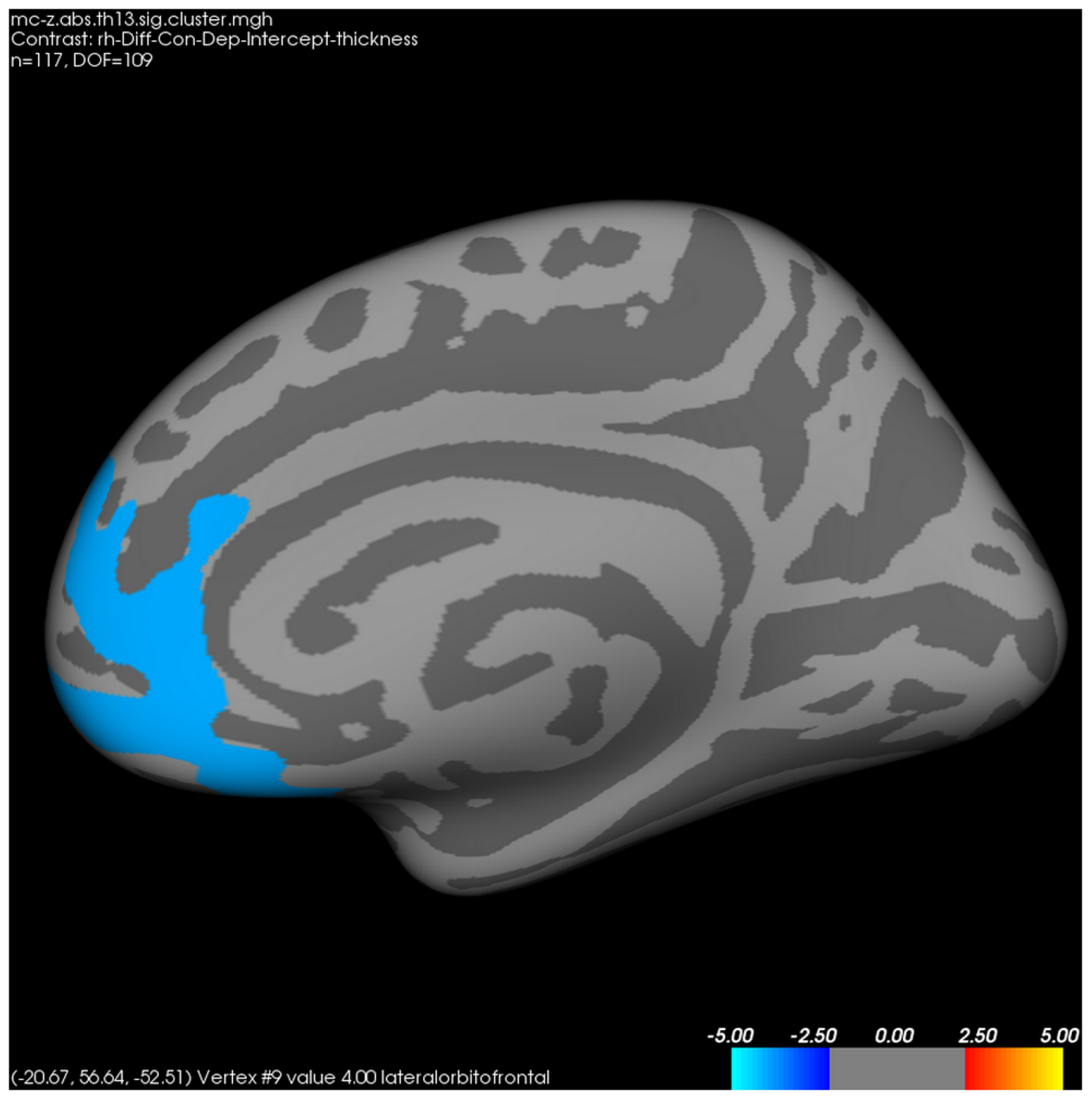
Differences in right cortical thickness between patients with major depressive disorder and healthy controls. *FWE*-corrected *p*<0.05 through Monte-Carlo permutation test.

In the whole-brain analysis for cortical thickness between patients with MDD and healthy controls, the former had significantly thinner left rostromiddle frontal (**Fig. 3**), right lateral orbitofrontal (**Fig. 4**), and right pars triangularis cortices than healthy controls.

### Correlations among *NR3C1* methylation, brain structures, and clinical variables

**Figure 5:**
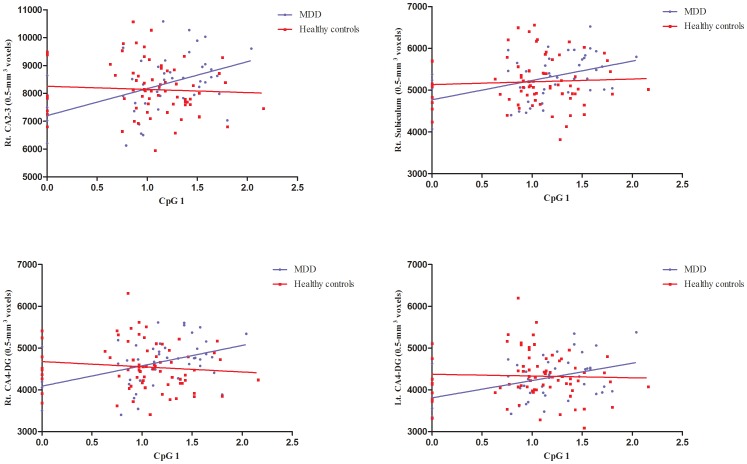
Correlations between *NR3C1* methylation at CpG1 and hippocampal subfield volumes among participants.

**Figure 6:**
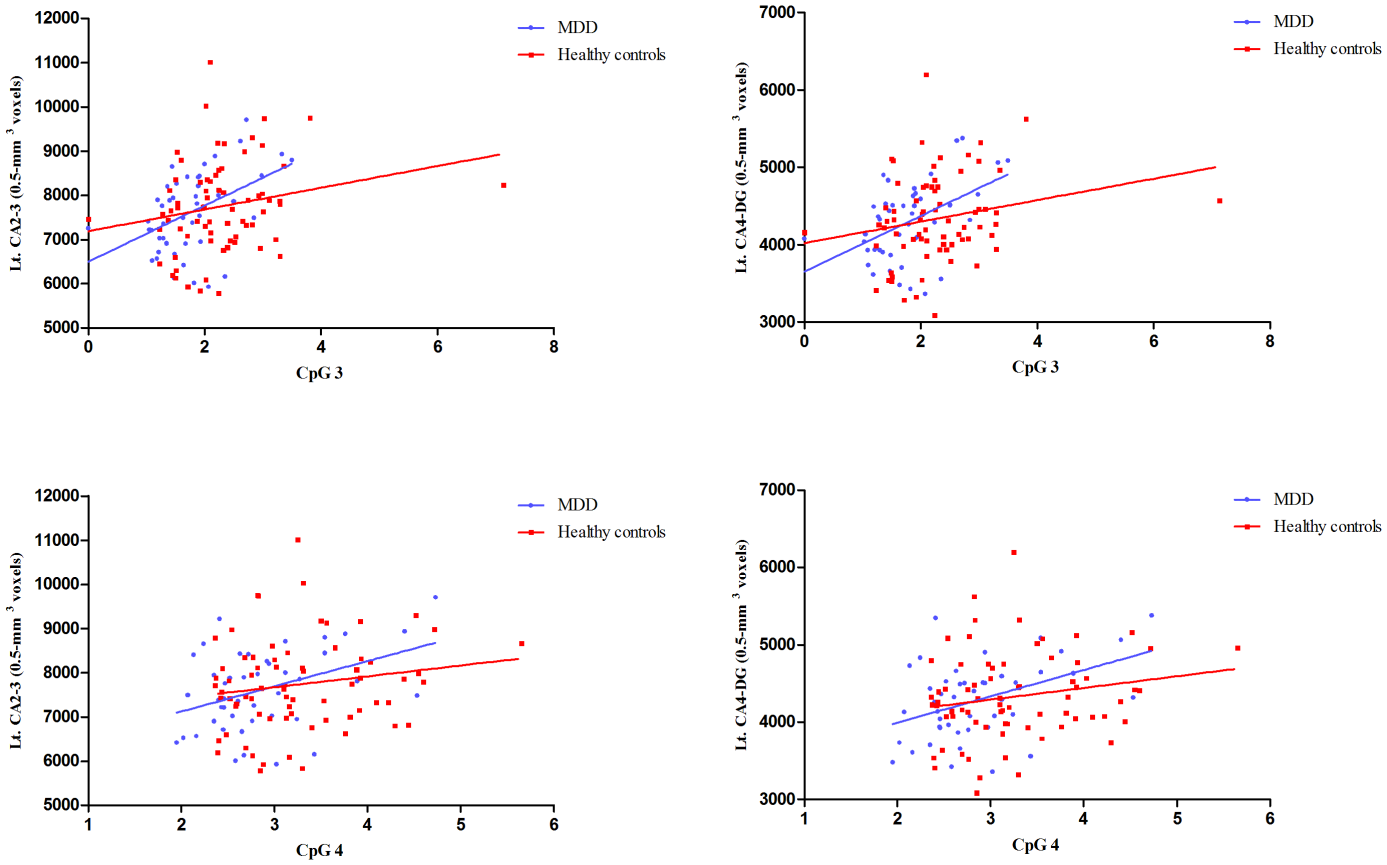
Correlations between *NR3C1* methylation at CpG2-3 and hippocampal subfield volumes among participants.

In the MDD group, methylation at CpG1 had positive correlations with total, right total, and left total hippocampal volumes, as well as right CA1 and left CA1 (**Fig. 5** **,** **Fig. 6** **, and Table S3 in File S1**). Methylation at CpG3 and CpG4 had positive correlations with left CA2-3, left CA4-DG, and left hippocampal fissure volumes. Methylation at CpG2 and CpG5 had no correlations with hippocampal volumes. In healthy controls, there was no relationship between methylation and total hippocampal volume. Methylation at CpG2 had positive correlations with left fimbria volume. Methylation at CpG 3 and CpG4 had positive correlations with left presubiculum and subiculum volumes. Methylation at CpG1, CpG2, and CpG5 had no correlations with hippocampal subfield volumes. Duration of illness was negatively correlated with right total hippocampal volumes, as well as right CA1, right CA2-3, right CA4-DG, right subiculum, and right fissure volumes. However, there were no relationships between duration of illness and left hippocampal volumes (**Table S4 in File S1**). Perceived stress had negative correlations with methylation at CpG3 in patients with MDD and CpG5 in healthy controls (**Table S5 in File S1**). Duration of illness was negatively correlated with methylation at CpG2 in patients with MDD.

In the whole-brain analysis for cortical thickness, duration of illness had positive associations with right isthmus (*r* = 0.442, *p* = 0.004) and right supramarginal (*r* = 0.351, *p* = 0.024) cortical thickness. In healthy controls, methylation at CpG1 had associations with the left pars orbitalis (*r* = −0.318, *p* = 0.008) and left posterior cingulate cortex (*r* = −0.288, *p* = 0.016). CpG4 was associated with the left fusiform cortex (*r* = −0.242, *p* = 0.045). CpG5 had associations with the left rostromiddle frontal (*r* = −0.261, *p* = 0.031) and right precentral cortex (*r* = 0.277, *p* = 0.021).

In patients with MDD, methylation at CpG1 was associated with the left entorhinal cortex (*r* = 0.327, *p* = 0.037). CpG2 was associated with the left frontal polar cortex (*r* = 0.331, *p* = 0.034). CpG3 was associated with the right pars triangular cortex (*r* = −0.316, *p* = 0.044). CpG4 was associated with the left entorhinal cortex (*r* = 0.344, *p* = 0.028). CpG5 was associated with the left inferior parietal cortex (*r* = −0.330, *p* = 0.035). There were no significant association between CpG5 methylations and cortical thickness in healthy controls.

## Discussion

To the best of our knowledge, this is the first study to compare *NR3C1* promoter region methylation between patients with MDD and healthy controls. We found a significant relationship between *NR3C1* promoter methylation and hippocampal volumes in MDD patients. However, contrary to our hypothesis, our patients with MDD had significantly lower methylations at the CpG3 and CpG4 than healthy controls. There were no CpG sites in which methylation was increased in patients with MDD compared to healthy controls. Previous studies have reported that higher methylation mat be related to repressed *NR3C1* transcription, impaired glucocorticoid receptor function, and consequently HPA axis hyperactivity in MDD [9], [11], [25]. Also, as described in the introduction section, most of the previous studies focused on the relationship between *NR3C1* methylation and early life adversities [12], [13], [32], [33], rather than clinical status. When considering the subjects of these previous studies, higher methylation was found not only in clinical samples such as suicide victims [12] and borderline personality disorder patients [13], but also in healthy controls [33] and in a community-based general population [32]. Thus, there is a possibility that higher *NR3C1* methylation found in these previous studies might represent a ‘scar’ of past childhood adversity, rather than current psychiatric vulnerability.

There were positive relationships between methylation and hippocampal subfield volumes in patients with MDD. NR3C1 methylation at CpG sites and increase in hippocampus subfield volumes were significantly associated in a greater number of regions, in patients with MDD compared to healthy controls. The degree of statistical significance was also greater in patients with MDD than in healthy controls. These results are not surprising, because glucocorticoid receptors are mainly involved in situations in which glucocorticoid levels are high, whereas mineralocorticoid receptors mediate glucocorticoid at a physiological level [34]. Methylations were particularly closely associated with CA2-3 and CA4-DG in patients with MDD. CA3, CA4, and DG are well known to be vulnerable to stress, thereby leading to depression and memory impairment [35]–[37]. DG and CA3 form neuronal circuitry mediated by mossy fibers, and are involved in synaptic and dendritic alterations under chronic stress, along with alterations in glucocorticoid activity [38], [39].

Although glucocorticoid receptor resistance and impaired negative feedback loop of HPA hyperactivity is one of the major neuroendocrine alterations in MDD, the results have not been consistently replicated. Rather, HPA hyperactivity has been mainly found in psychotic depression [40] or severe depression [41]. A previous study reported that non-psychotic patients with MDD showed decreased 24 hour cortisol secretion, whereas psychotic patients with MDD had increased 24 hour cortisol secretion compared to healthy controls [7]. A recent study also reported that mild to moderate degree of depression might be associated with enhanced glucocorticoid receptor function and decreased HPA axis function [8]. In a meta-analysis, non-psychotic depressive patients had a 41% non-suppression for dexamethasone suppression test (DST) whereas psychotic patients had a 64% non-suppression [40]. One randomized controlled study even reported that intravenous hydrocortisone improved depressive symptoms [42]. Similar to the characteristics of depressive patients in the above studies, most of the patients in our study were diagnosed with mild to moderate depression. Thus, the higher *NR3C1* methylation raises a possibility that methylation occurs in a compensatory pattern. However, given our study design and results, we could not determine whether the HPA axis was hypoactive in our MDD sample. Further studies should focus on the possible intrinsic mechanisms and effects of *NR3C1* methylation in relation with HPA axis in MDD.

On the other hand, we could not find differences in hippocampal subfield volumes between patients with MDD and healthy controls. Whereas initial studies reported that chronic exposure to glucocorticoid could result in neuronal loss of the hippocampus [43], subsequent studies failed to consistently replicate the deleterious effects of glucocorticoid on the hippocampus [44], [45]. Contrary to the non-significant differences in hippocampal volumes, in the whole brain analysis for cortical thickness, patients with MDD had significantly thinner left rostromiddle frontal, right lateral orbitofrontal, and right pars triangularis cortex compared to healthy controls. These results are in accordance with a previous study reporting that orbitofrontal cortex was significantly decreased in patients with MDD (n = 23) compared to healthy controls (n = 26) [46], although no significant relationship between *NR3C1* methylations and cortical thickness was reported.

Finally, although the significance level was relatively small, our findings of negative correlations between methylations and perceived stress supports the notion that methylation may occur to compensate the harmful alterations that are produced during the course of depressive episodes. Whereas the environment and maternal care are main determinants for stress in animals and humans during the early childhood period, adulthood stress response is determined in the context of cognitive appraisal of individuals who perceive the stress [47], [48]. Thus, it is worth to further investigate the causal relationship between adulthood perceived stress and epigenetic alterations.

There are several limitations that should be mentioned. First, we did not measure neuromolecular and biological factors that are possibly associated with the regulation and function of glucocorticoid receptors, which made it difficult to interpret neuromolecular mechanisms underlying associations between *NR3C1* methylations and hippocampal subfield volumes. Second, we did not consider the childhood abuse history of participants, contrary to previous studies, since our main focus was to investigate whether the methylation in *NR3C1* is different between patients with MDD and healthy controls. However, since we did not measure childhood adversities, we could not differentiate subgroups with childhood adversities. Our results, such as a lower *NR3C1* methylation of patients with MDD and a positive correlation between *NR3C1* methylation and hippocampal subfield volumes, might reflect clinical characteristics of MDD patients who do not have childhood adversities. Retrospective measures of childhood adversity are inevitably associated with recall bias. In addition, due to financial constraints and ethical issues, it is difficult to prospectively measure since childhood, the associations between childhood adversity and *NR3C1* methylation, as well as HPA axis alterations.Finally, we measured *NR3C1* methylation in peripheral blood, but not hippocampal tissues. Several studies have reported that methylation occurs in a tissue-specific manner [12], [49]. Since DNA methylation is dependent on different brain regions, there is a possibility that our data represent *NR3C1* distributed throughout various brain regions, and not confined to the hippocampus. However, previous studies reported that peripheral *NR3C1* gene promoter methylation was associated with central glucocorticoid receptor mRNA expression [13], [25]. Since peripheral factors representing the HPA axis, such as cortisol awakening response, could have a relationship with structural brain abnormalities including the hippocampus [50], [51], *NR3C1* methylation might also have correlations with hippocampal subfield volumes and cortical thickness. Additionally, considering its convenience for repeated measures and longitudinal follow-up, peripheral methylation has been recommended as a candidate method for measuring methylation in humans [52].

Despite the above limitations, our study has several clinical implications. First, to the best of our knowledge, this is the first study to compare *NR3C1* gene methylation between patients with MDD and healthy controls. Second, by measuring hippocampal subfield and cortical thickness, we further comprehensively examined the association between methylation and structural brain abnormalities. Particularly, we strictly recruited drug-naïve and non-psychotic patients with MDD to avoid possible confounding effects for methylation and structural brain abnormalities. Third, we used automated segmentation of hippocampal subfield volumes, which enabled us to measure hippocampal subfields with distinct functional characteristics. Specifically, regarding CA2-3 and CA4-DG subfields, the correlation coefficients with manual segmentation were 0.91 and 0.82, respectively [30].

In conclusion, through this study, we raised critical issues regarding the role of *NR3C1* methylation in patients with MDD as compared to healthy controls. Future studies with longitudinal designs and comprehensive measures of genetic and neurobiological factors related to *NR3C1* methylation should be conducted in individuals with MDD.

## Supporting Information

File S1 **Table S1, Comparison of methylation between patients with major depressive disorder and healthy controls. Table S2, Mean hippocampal subfield volumes (measured in 0.5-mm^3^ voxels) between patients with major depressive disorder and healthy controls. Table S3, Partial coefficients between methylation and hippocampal subfield volumes among patients with major depressive disorder and healthy controls. Table S4, Correlations between duration of illness and hippocampal subfield volumes among patients with major depressive disorder. Table S5, Partial coefficients between methylation and clinical variables in participants with major depressive disorder and healthy controls. Figure S1, Schematic representation of NR3C1 promoter regions assessed by pyrosequencing.**
(DOCX)
