# Supplementary material for: Association between Glucocorticoid Receptor Methylation and Hippocampal Subfields in Major Depressive Disorder
Source: PLoS One. 2014 Jan 21;9(1):e85425. doi: 10.1371/journal.pone.0085425 (PMC3897456; doi:10.1371/journal.pone.0085425)
Supplement: File S1 — Table S1, Comparison of methylation between patients with major depressive disorder and healthy controls. Table S2, Mean hippocampal subfield volumes (measured in 0.5-mm3 voxels) between patients with major depressive disorder and healthy controls. Table S3, Partial coefficients between methylation and hippocampal subfield volumes among patients with major depressive disorder and healthy controls. Table S4, Correlations between duration of illness and hippocampal subfield volumes among patients with major depressive disorder. Table S5, Partial coefficients between methylation and clinical variables in participants with major depressive disorder and healthy controls. Figure S1, Schematic representation of NR3C1 promoter regions assessed by pyrosequencing. (DOCX) [file pone.0085425.s001.docx]

**Table S1. Comparison of methylation between patients with major depressive disorder and healthy controls**

|  | **MDD** | | **Healthy controls** | | ***F*** | ***p*** |
| --- | --- | --- | --- | --- | --- | --- |
| **CpG1** | 1.15 | (0.48) | 1.01 | (0.48) | 1.641 | 0.198 |
| **CpG2** | 3.14 | (0.72) | 3.37 | (1.11) | 1.222 | 0.299 |
| **CpG3** | 1.76 | (0.66) | 2.24 | (0.92) | 6.266 | 0.003 |
| **CpG4** | 2.86 | (0.64) | 3.26 | (0.70) | 6.260 | 0.003 |
| **CpG5** | 1.04 | (0.60) | 1.09 | (0.70) | 0.081 | 0.923 |

All data are represented as mean (*SD*).

Results were adjusted for age and sex.

**CpG1:** -293, **CpG2:** -286, **CpG3:** -283, **CpG4:** -277, **CpG5:** -274. Distance (nt) from transcription start site (+1).

**Table S2. Mean hippocampal subfield volumes (measured in 0.5-mm^3^ voxels) between patients with major depressive disorder and healthy controls**

|  | **MDD** | | **Healthy controls** | | ***F*** | ***p*** |
| --- | --- | --- | --- | --- | --- | --- |
| **Total** | 49991.12 | (4871.95) | 49470.03 | (5334.87) | 3.204 | .076 |
| **Rt. Total** | 25736.21 | (2642.49) | 25009.84 | (2769.73) | 5.257 | .024 |
| **Lt. Total** | 24254.91 | (2368.19) | 24227.08 | (2803.80) | .630 | .429 |
| **Rt. CA1** | 2844.83 | (380.92) | 2777.01 | (349.50) | 2.348 | .129 |
| **Rt. CA2-3** | 8312.75 | (1047.61) | 8139.96 | (923.60) | 4.272 | .041 |
| **Rt. CA4-DG** | 4650.51 | (558.28) | 4552.94 | (552.39) | 3.181 | .077 |
| **Rt. Fimbria** | 488.93 | (154.81) | 489.93 | (149.65) | .762 | .385 |
| **Rt. Presubiculum** | 3781.63 | (425.11) | 3640.84 | (475.03) | 6.728 | .011 |
| **Rt. Subiculum** | 5303.36 | (527.34) | 5201.23 | (577.95) | 2.949 | .089 |
| **Rt. Fissure** | 354.21 | (164.20) | 325.68 | (151.64) | 1.157 | .285 |
| **Lt. CA1** | 2638.85 | (280.12) | 2700.22 | (365.24) | .198 | .658 |
| **Lt. CA2-3** | 7615.23 | (908.48) | 7738.06 | (1026.73) | .000 | .993 |
| **Lt. CA4-DG** | 4286.61 | (499.01) | 4332.50 | (568.82) | .081 | .777 |
| **Lt. Fimbria** | 537.64 | (168.43) | 551.96 | (165.98) | .019 | .890 |
| **Lt. Presubiculum** | 3716.66 | (426.42) | 3584.55 | (461.00) | 8.877 | .004 |
| **Lt. Subiculum** | 5184.16 | (553.06) | 5152.62 | (555.22) | 2.010 | .159 |
| **Lt. Fissure** | 275.76 | (101.71) | 282.52 | (108.29) | .041 | .840 |

All data are represented as mean (*SD*).

*Df* = 2

Results were adjusted for age, sex, and intracranial volume.

**Table S3. Partial coefficients between methylation and hippocampal subfield volumes among patients with major depressive disorder and healthy controls**

| **Major depressive disorder** | | | | | | | | | | | | | | | | |
| --- | --- | --- | --- | --- | --- | --- | --- | --- | --- | --- | --- | --- | --- | --- | --- | --- |
|  |  |  | **Rt hippocampal subfield** | | | | | | | **Lt. hippocampal subfield** | | | | | | |
| **Total** | **Rt. total** | **Lt. total** | **CA1** | **CA2-3** | **CA4-DG** | **Fimbria** | **Presubiculum** | **Subiculum** | **Fissure** | **CA1** | **CA2-3** | **CA4-DG** | **Fimbria** | **Presubiculum** | **Subiculum** | **Fissure** |
| **.364^*^** | **.418^†^** | **.445^†^** | **.321^*^** | .280 | -.149 | -.013 | .281 | .198 | .261 | **.306^*^** | .265 | .280 | -.057 | .101 | .220 | .107 |
| .170 | .214 | .099 | .108 | .090 | -.049 | -.041 | .097 | .024 | .106 | -.037 | .190 | .137 | .040 | .034 | -.056 | .227 |
| .136 | .167 | .236 | .161 | .214 | -.142 | .096 | .122 | **.313^*^** | .087 | .145 | **.385^*^** | **.413^†^** | .108 | .174 | .162 | **.416^†^** |
| .304 | **.311^*^** | .268 | .180 | .230 | -.178 | .198 | .223 | .255 | .253 | .239 | **.433^†^** | **.471^†^** | -.066 | .195 | .247 | **.378^*^** |
| -.004 | .064 | .242 | .151 | .154 | .125 | -.039 | .119 | .196 | -.065 | .014 | .091 | .112 | -.025 | -.023 | .011 | .291 |
| **Healthy controls** | | | | | | | | | | | | | | | | |
|  |  |  | **Rt hippocampal subfield** | | | | | | | **Lt. hippocampal subfield** | | | | | | |
| **Total** | **Rt. total** | **Lt. total** | **CA1** | **CA2-3** | **CA4-DG** | **Fimbria** | **Presubiculum** | **Subiculum** | **Fissure** | **CA1** | **CA2-3** | **CA4-DG** | **Fimbria** | **Presubiculum** | **Subiculum** | **Fissure** |
| .075 | .035 | -.037 | .092 | .025 | .179 | .073 | .149 | -.049 | .103 | .053 | .109 | .106 | .056 | .123 | .148 | .014 |
| .116 | .085 | -.015 | .011 | .079 | -.066 | .064 | .094 | -.142 | .126 | -.051 | -.018 | .005 | .178 | .127 | .011 | -.099 |
| .103 | .174 | .080 | .214 | .149 | .181 | .234 | .219 | -.096 | .005 | .063 | .132 | .148 | .037 | **.310^†^** | **.282^*^** | -.113 |
| .057 | .100 | .061 | .077 | .068 | .040 | **.283^*^** | .228 | -.051 | .000 | .131 | .067 | .088 | .046 | **.308^*^** | **.240^*^** | -.055 |
| .058 | .016 | -.077 | -.014 | -.080 | .047 | .136 | .013 | -.018 | .092 | -.173 | -.083 | -.080 | -.091 | .111 | -.073 | .013 |

**p* < 0.05, ^†^*p* < 0.005, *df* = 40 for major depressive disorder and 67 for healthy controls.

Partial coefficients were adjusted for age, sex, and intracranial volume.

**Table S4. Correlations between duration of illness and hippocampal subfield volumes among patients with major depressive disorder**

|  |  |  |  | **Rt hippocampal subfield** | | | | | | | **Lt. hippocampal subfield** | | | | | | |
| --- | --- | --- | --- | --- | --- | --- | --- | --- | --- | --- | --- | --- | --- | --- | --- | --- | --- |
|  | **Total** | **Rt. total** | **Lt. total** | **CA1** | **CA2-3** | **CA4-DG** | **Fimbria** | **Presubiculum** | **Subiculum** | **Fissure** | **CA1** | **CA2-3** | **CA4-DG** | **Fimbria** | **Presubiculum** | **Subiculum** | **Fissure** |
| ***r*** | -.318 | -.330 | -.361 | -.393 | -.402 | .138 | .085 | -.378 | -.400 | -.259 | -.227 | -.259 | -.281 | .018 | -.020 | -.211 | -.247 |
| ***p*** | .040 | .033 | .019 | .010 | .008 | .382 | .593 | .014 | .009 | .098 | .149 | .098 | .071 | .912 | .898 | .180 | .114 |

Adjusted for age, sex, and intracranial volume

**Table S5. Partial coefficients between methylation and clinical variables in participants with major depressive disorder and healthy controls**

|  | **Perceived stress** | | | | **HRSD** | | **Duration of illness** | |
| --- | --- | --- | --- | --- | --- | --- | --- | --- |
|  | **MDD** | | **Healthy controls** | |  |  |  |  |
|  | ***r*** | ***p*** | ***r*** | ***p*** | ***r*** | ***p*** | ***r*** | ***p*** |
| **CpG1** | .022 | .890 | -.030 | .804 | -.172 | .271 | -.216 | .164 |
| **CpG2** | .075 | .631 | -.025 | .839 | -.126 | .419 | **-.357** | .019 |
| **CpG3** | **-.310** | .043 | .209 | .083 | .041 | .795 | -.050 | .748 |
| **CpG4** | -.076 | .629 | .199 | .099 | .193 | .216 | -.118 | .451 |
| **CpG5** | -.338 | .027 | **-.250** | .037 | -.295 | .055 | -.222 | .153 |

Adjusted for age and sex

MDD: major depressive disorder, HRSD: Hamilton Rating Scale for Depression

**Figure S1. Schematic representation of NR3C1 promoter regions assessed by pyrosequencing**

**
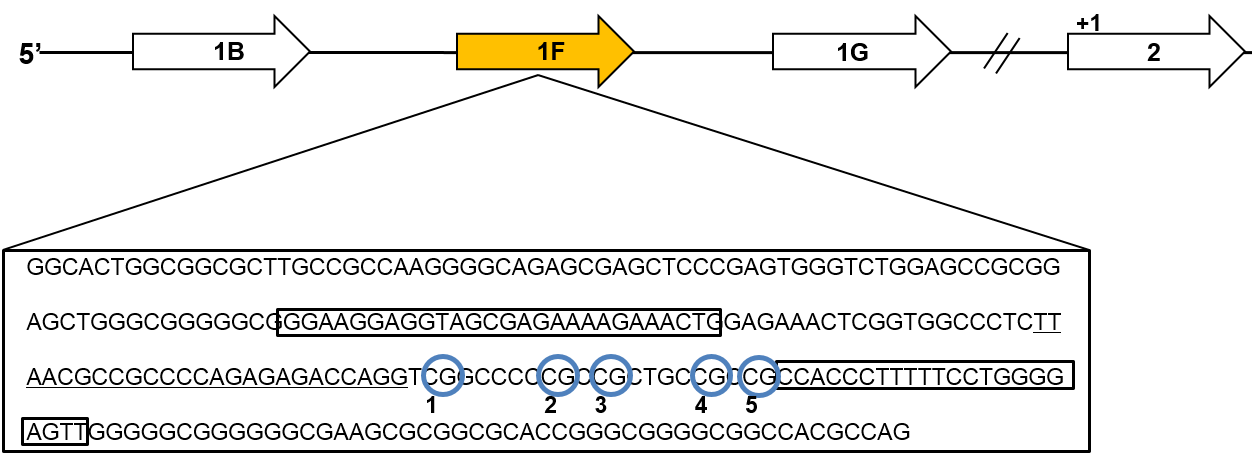
**

The region from nucleotide -293 to -274 (distance (nt) from transcription start site (+1)) of the 5’-end of the NR3C1 gene. Underlined: sequencing primer; square boxes: position of the oligonucleotides used for amplification of bisulfite-treated DNA; encircled: CpGs analyzed.
